# A new low-cost negative-pressure wound therapy *versus* a commercially available therapy device widely used to treat complex traumatic injuries: a prospective, randomized, non-inferiority trial

**DOI:** 10.6061/clinics/2017(12)04

**Published:** 2017-12

**Authors:** Fabio Kamamoto, Ana Lucia Munhoz Lima, Marcelo Rosa de Rezende, Rames Mattar-Junior, Marcos de Camargo Leonhardt, Kodi Edson Kojima, Carla Chineze dos Santos

**Affiliations:** I Pesquisador, Hospital das Clinicas HCFMUSP, Faculdade de Medicina da Universidade de Sao Paulo, Sao Paulo, SP, BR; II Departamento de Controle de Infeccao Hospitalar, Hospital das Clinicas HCFMUSP, Faculdade de Medicina da Universidade de Sao Paulo, Sao Paulo, SP, BR; III Departamento de Microcirurgia, Hospital das Clinicas HCFMUSP, Faculdade de Medicina da Universidade de Sao Paulo, Sao Paulo, SP, BR; IV Instituto de Ortopedia e Traumatologia, Hospital das Clinicas HCFMUSP, Faculdade de Medicina da Universidade de São Paulo, São Paulo, SP, BR; V Consultor Independente, Sao Paulo, SP, BR

**Keywords:** Negative-Pressure Wound Therapy, Wound Healing, Wounds and Injuries, Cost Savings

## Abstract

**OBJECTIVES:**

Negative-pressure wound therapy has been widely adopted to reduce the complexity of treating a broad range of acute and chronic wounds. However, its cost is high. The objective of this study was to evaluate the following two different methods of negative-pressure wound therapy in terms of healing time: a low-cost method of negative-pressure wound therapy (a pressure stabilizer device connected to a hospital wall-vacuum system with a gauze-sealed dressing, USP) and the standard of care (vacuum-assisted closure, VAC).

**METHODS:**

This is a randomized, controlled, non-inferiority, unblinded trial. Patients admitted with complex injuries to a trauma center in a public referral hospital who were indicated for orthopedic surgery were randomized to a USP or VAC group. The primary outcome was the time required to achieve a “ready for surgery condition”, which was defined as a wound bed with healthy granulation tissue and without necrosis or purulent secretion. Wound bed area contraction, granulation tissue growth and the direct costs of the dressings were secondary outcomes.

**RESULTS:**

Variation in area and granulation tissue growth were essentially the same between the systems, and healing time was equal between the groups (*p*=0.379). In both systems, serial debridement increased wound area (*p*=0.934), and granulation tissue was also increased (*p*=0.408). The mean treatment cost was US$ 15.15 in the USP group and US$ 872.59 in the VAC group.

**CONCLUSIONS:**

For treating complex traumatic injuries, USP was non-inferior to and less expensive than VAC.

## INTRODUCTION

Negative-pressure wound therapy (NPWT) was approved by the FDA (Food and Drug Administration) in 1996 and has since then been widely adopted as a treatment for a broad range of wounds 1. NPWT has many indications, including both acute and chronic injuries 2, and has simplified the treatment of wounds for doctors, and nurses 2.

Some authors have suggested that NPWT could be used in traumatic wounds as a bridge to definitive closure when primary closure is not possible after or between debridement procedures 3-5. The most substantial disadvantage of this procedure is its cost, which remains high and generally unaffordable over prolonged use 2,6. As mentioned by the authors of a University of Chicago Medical Center (UCMC) study: “this financial burden can limit the use of NPWT in settings and situations where budgets are constrained, particularly in public hospitals and for patients who are underinsured or uninsured (not to mention in developing countries globally). From the health care providers’ standpoint, our interest in coming up with more cost-effective alternatives is obvious; such efforts can ultimately translate to increased availability/accessibility of therapies for patients in various settings. Furthermore, for issues as prevalent as wound care management, they can also have a significant financial impact on hospitals and the health care system as a whole” 7. In 2007, the University of São Paulo (USP) developed a low-cost NPWT that used a pressure stabilizer device (Curavac VX 200, Ventrix Health Innovation, Brazil), connected to a hospital wall-vacuum and a gauzed-sealed dressing. This system will henceforth be referred to as “USP”. However, so far, no randomized clinical trial has compared USP NPWT with a commercially available vacuum-assisted closure device (VAC system, KCI, San Antonio, TX, USA). We therefore performed the present study.

Although different NPWT systems are available, VAC was chosen as the standard of care based on feasibility. The VAC system was the first NPWT to be approved by the FDA since 1996, and it remains the leader in sales worldwide and the only one available in the University of São Paulo Hospital.

The primary objective was to determine whether the “USP” method is non-inferior to the standard of care, VAC, with respect for the time required to achieve a “ready for surgery” status in trauma patients.

The following were secondary outcomes: changes in wound size, granulation tissue growth and the overall associated costs of the dressings between the two systems.

## METHODS

### Design, setting and ethics

A prospective, randomized, non-inferiority trial was designed to compare two different negative-pressure devices used for wound closure. The trial occurred at the Orthopedics and Traumatology Institute of Hospital das Clínicas, Faculdade de Medicina, Universidade de São Paulo (FMUSP), in São Paulo, Brazil. The study was previously approved by the University of São Paulo Ethics Committee (0371/11), and the protocol is registered in the ClinicalTrials.gov Registry.

### Participants and sampling

All consecutive patients admitted to one public university hospital (Hospital das Clínicas, University of São Paulo) with acute wounds caused by open fractures inflicted by high-energy trauma between September 2013 and July 2016 were eligible for this study. To be included in this study, the cases had to involve severe wounds that would not close on the first attempt (without surgery).

The exclusion criteria were diabetes mellitus, diagnosed peripheral vascular disease, chronic use of steroids, coagulopathies and cancer.

The sample size was calculated to allow a comparison of the time required to achieve a wound bed that is ready for surgery. Since we expected USP and VAC to have similar effects on wound bed preparation time, we used a non-inferiority testing strategy. The non-inferiority margin was set at 5%, meaning that the data would support the non-inferiority of USP if the number of days necessary to be ready for surgery was no more than 5% greater in the USP than in the VAC group. To achieve 80% statistical power and a significance (alpha) level of 5%, the estimated required sample size was 36 treatments in each group.

### Interventions

In the VAC group, GranuFoam sponge (Kinetics Concepts, Inc., San Antonio, TX, USA) was applied to the wounds and sealed using an occlusive plastic dressing. Continuous suction at 120 mmHg was then initiated.

In the USP group, a 100% cotton sterile gauze dressing was applied to the wound. Then, an 18-Fr polyvinyl nasogastric tube was placed in the center of the dressing and sealed in place using a transparent adhesive and a sterile, waterproof film (Opsite Drape, Smith & Nephew, Kingston Upon Hill, UK). The dressing was connected to the hospital’s continuous suction equipment that was accessed on the wall of each room via a plastic sterile secretion collector tube. An anti-bacterial filter was used to avoid contamination by the hospital vacuum net (Zammi, Rio de Janeiro, Brazil). Because the pressure in the vacuum net is naturally unstable, a pressure stabilizer device was specifically designed for this purpose (Curavac VX 200, Ventrix Health Innovation, Brazil). The equipment was regulated to provide a continuous pressure of 120 mmHg. This device uses a system of springs to restrain any increase in pressure over the established limit and keep it stable when wall vacuum pressure decreases.

The dressing was changed twice per week in all patients while they were under general anesthesia. When necessary, surgical debridement was performed at the same time. During dressing changes, all disposable articles were discarded. The pressure stabilizer device that was used in the study group is composed of a permanent material that can be used for many treatments.

### Outcomes

The primary outcome used was the time (in days) necessary to achieve a “ready for the surgery” condition, which was defined as a wound bed with healthy granulation tissue and without necrosis or purulent secretion 8.

The patient follow-up period started as soon as the first debridement was performed and the NPWT dressings were placed and continued until a surgery achieved definitive wound closure.

The following were secondary outcomes:

1. **Changes in wound bed area over time**. The dimensions of each wound were documented using digital photography at each dressing change. The images were uploaded and the wound area calculated using the National Institutes of Health ImageJ software with the scale set using the ruler in the image.
2. **Granulation tissue growth:** The percentage of the wound bed that was composed of granulation tissue was calculated in wound images by dividing the amount of red tissue area by the total wound area.
3. **Costs:** The direct costs of each type of dressing were also measured. In both groups, these costs included the cost of supplies (e.g., suction canisters, catheters or drains, tubing, gauze, and adhesive drapes). The following two main cost analyses were performed: the costs associated with each dressing exchange and the average cost of treatment. The direct costs of dressing exchange were recorded (i.e. the cost of inputs) for each of the treatments applied in the USP and VAC groups. In the USP group, the prices paid by the hospital for a 55 x 45 cm sterile adhesive film, a zoobec sterile gauze, a urethral catheter no. 16 and a 500 ml secretion collecting bottle were recorded. In the VAC group, the costs of the sponge kit and the secretory collection bottle were recorded. To determine the average cost of treatment, the cost of each dressing change was multiplied by the number of exchanges performed in each of the groups during the study period. The prices were quoted on October 21, 2016 and obtained from the hospital’s electronic stock control system.

### Failure of intervention

Failure of therapy was recorded in any of the following situations:

1. A dressing could not be maintained because of persistent fluid or suction leaks. If, after two attempts at dressing reinforcement during a 24-hour period, the seal could not be maintained, the patient was considered a case of intervention failure.
2. If a patient developed bleeding, invasive wound infection or sepsis, or a situation in which the dressing could worsen the patient’s clinical condition, NPWT was discontinued and considered a failure.
3. If the patient died before the achieving a “ready for surgery” condition.

### Randomization and blinding

To allocate the participants, a computer-generated list was obtained using the website www.randomization.com. The patients were randomized by the number sequence to either the USP or VAC group in a 1:1 ratio in four blocks. The allocations were printed and inserted into opaque, sealed, numbered envelopes. A research assistant who was blind to the allocation drew one envelope per consecutive patient and then revealed the allocation to the assistant physician only at the moment the patient was admitted for therapy.

The two methods used to perform NPWT were obviously visually different, and it was therefore impossible to blind either the therapists or the patients to the method of treatment. However, the two researchers who used ImageJ to perform the measurements used to evaluate wound closure in each digital image were blinded to patient allocation.

### Statistical methods

A per protocol analysis was performed to evaluate the primary and secondary outcomes. Initially, all data were recorded descriptively, as absolute numbers, frequencies, and confidence intervals for categorical data and as the means and standard deviations for continuous data. Pearson’s chi-squared test was used for comparisons of categorical data, and Mann-Whitney or Student’s t tests were used for comparisons of continuous data between the groups. An alpha error of 0.05 was adopted as indicative of significance. SPSS 23 for Mac software was used for the statistical analysis.

## RESULTS

### Participant flow, treatment failures and baseline data

**Figure 1:**
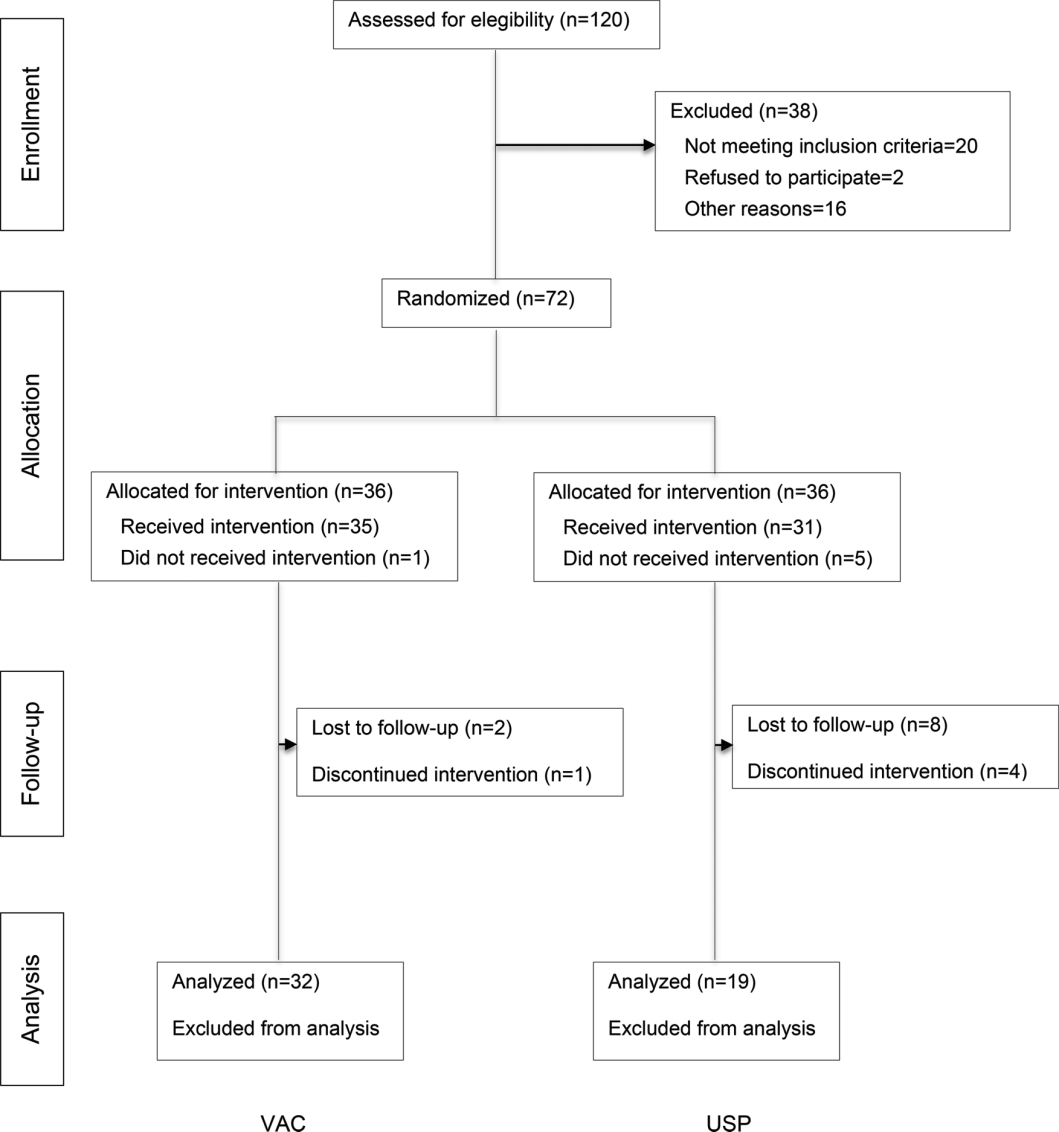
Flow diagram of patient inclusion and exclusion.

During the study period, 120 patients were assessed for eligibility. Of these, 38 were excluded (20 who did not meet eligibility criteria and 18 who were unable to consent). Two further patients refused to participate. As shown in the flow diagram presented in Figure 1, a total of 72 patients (36 in each group) were enrolled in the study. Baseline demographics and clinical data were similar between the groups and are listed in Table 1. A total of 19 wounds were analyzed in the USP group, and 32 were analyzed in the VAC group.

Two patients died in the USP group as a result of multiple organ failure during clinical care for severe trauma. No patient in the VAC group died. It was not possible to maintain vacuum pressure in 2 cases in the USP group and 1 case in the VAC group. The following patients were lost: in the USP group, 17 patients were lost due to: the hospital lacked the supplies required to perform the dressing procedure in the hospital (5 cases), the patient was transferred to another setting and was lost to follow-up (8 cases), and it was impossible to maintain vacuum (2 cases). In the VAC group, 2 cases were lost to follow-up, and in 1 case, it was impossible to maintain vacuum.

### Primary outcome: time to heal

**Figure 2:**
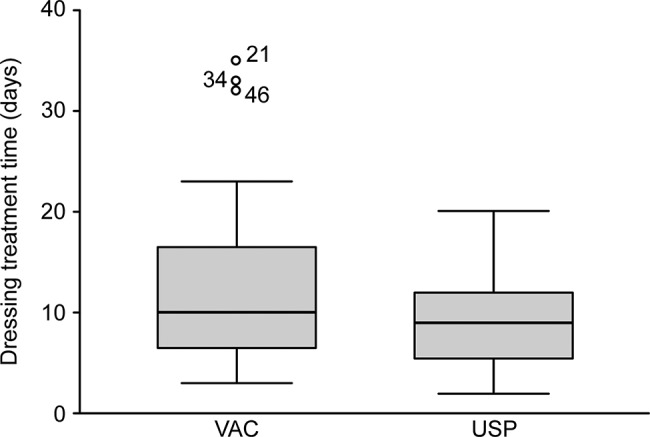
Time necessary to achieve a “ready for the surgery” condition (days).

There were 19 wounds in the 17 patients in the USP group, in which 15 had 1 wound, and 2 had 2 wounds each. The VAC group consisted of 30 patients with 1 wound and 1 patient with 2 wounds for a total of 32 wounds. All wounds in all patients were included in the analysis. The number of days necessary to achieve a “ready for surgery” condition was similar across the 51 included wounds. In the USP group, the 19 wounds took 9.6±4.5 days to heal, while in the VAC group, the 32 wounds took 12.8±8.6 days to recover (*p*=0.379) (Figure 2).

### Secondary outcomes

**Figure 3:**
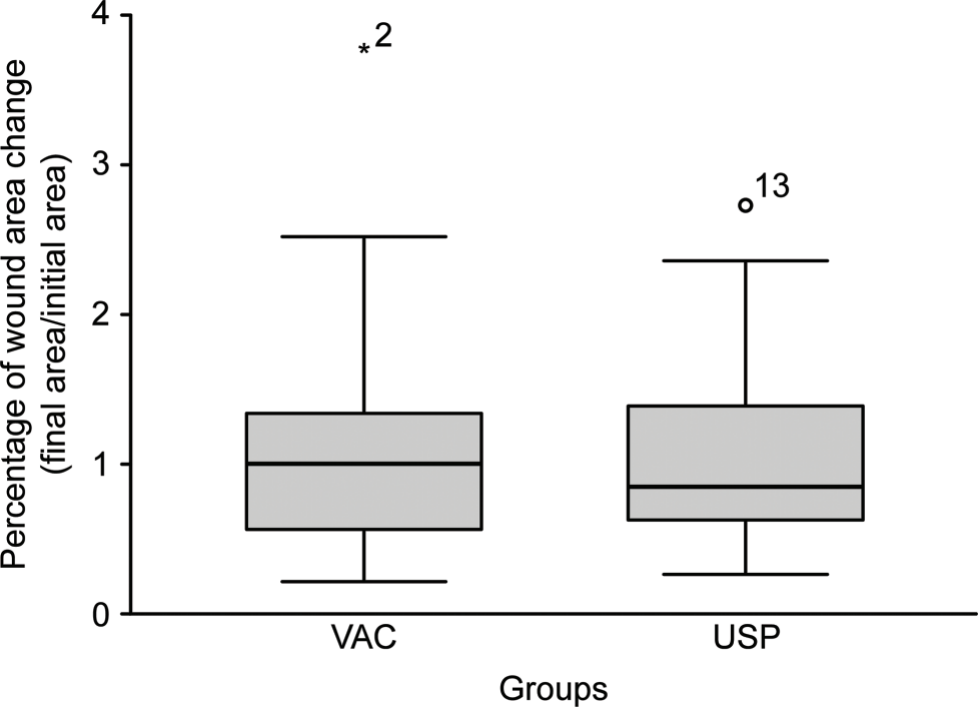
Changes in wound bed area (%).

**Figure 4:**
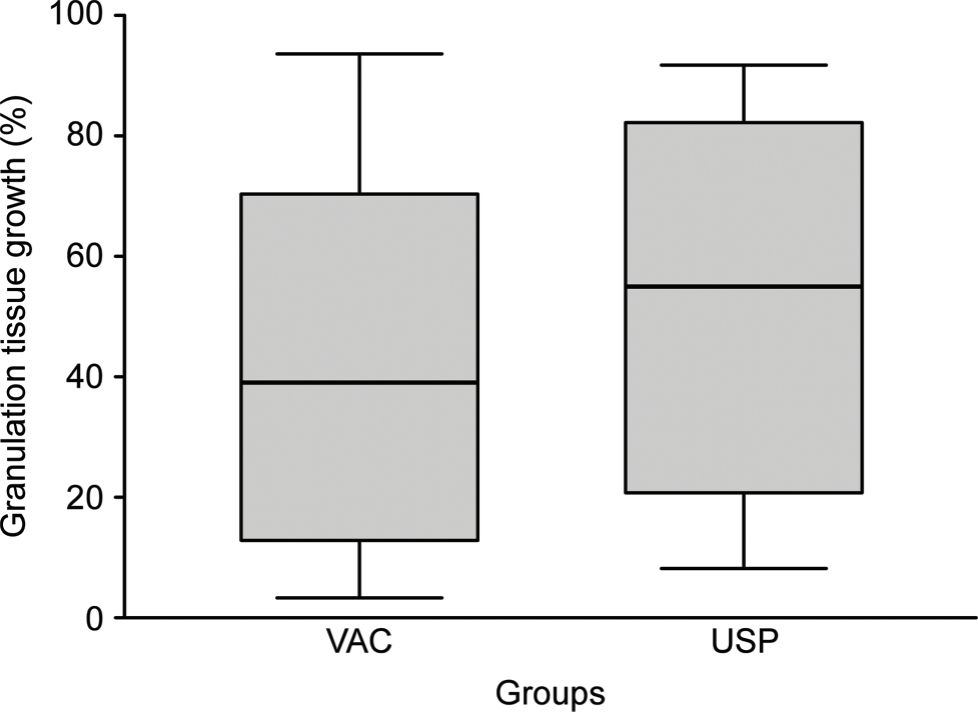
Granulation tissue increase (%).

1. **Changes in wound bed area:** there was an increase in wound surface area in both groups. However, the rate of change was not significantly different between the groups (1.13±0.80 in the USP group and 1.12±0.80 in the VAC group; *p*=0.934) (Figure 3).
2. **Granulation tissue growth:** There was an increase in granulation tissue percentage in both groups. The rate of change was 53.01% for USP and 44.18% for VAC. However, the rate of change between the groups was not significantly different (*p*=0.408). The percentage of granulation tissue growth per day for the USP and VAC groups were: 5.79±2.93 and 5.06±5.15) (*p*=0.408; Figure 4).
3. **Mean cost of treatment:**
  **Cost of each dressing:** The mean cost of supplies for each dressing used in VAC therapy was US$ 513.29 (R$ 1,622.00). The mean cost of supplies for each dressing changed performed in USP therapy was US$ 8.22 (R$ 26.00). These prices were quoted on October 21^st^ 2016 by the Purchasing Department of our hospital. The U.S. dollar exchange rate was 3.16 (as of October 21^st^, 2016).
  **Mean cost of treatment:** This metric was calculated as the mean cost of the dressings multiplied by the mean number of dressings applied. The total in the USP group was US$ 15.15 (R$ 47.89), and the total in the VAC group was US$ 872.59 (R$ 2,757.40). Tables 2 and 3 show that the cost was significantly different between the groups.

## DISCUSSION

Negative-pressure wound therapy (NPWT) was first described in 1966 9. Although various NPWT systems are currently available on the market, their costs have limited their accessibility in many institutions 2,6. More recently, researchers have studied low-cost methods of performing NPWT 2,6,10,11. In 2007, the University of São Paulo developed a low-cost NPWT based on a pressure stabilizer device. The experience of the authors with this method was published in 2010 as a case series 12. In the present study, we performed a prospective, randomized clinical trial to compare the USP method with another NPWT method that uses the VAC system.

We chose to perform a non-inferiority trial based on the expectation that the time necessary for the wound bed to improve (in days) would be longer in patients treated with USP therapy than in those treated with a VAC System (primary outcome). Wound bed area contraction and granulation tissue growth were also assessed as secondary outcomes. The USP group did indeed heal three days faster. Although this was not a statistically significant difference, the USP group presented less variability than the VAC group. In addition, USP and VAC were demonstrated to be equally effective, showing that the USP system is non-inferior to the VAC system, which is significantly more expensive. In a public trauma center such as ours, this difference can result in huge savings in resources that can therefore be used to treat more patients. The cost of treatment in the USP group was approximately 2% of the cost in the VAC system.

This study replicates and extends the results of Dorafshar et al. 6 and demonstrates that the two methods of NPWT (USP and VAC) presented here achieved very similar results with regard for increased granulation tissue. The wound area was increased in both groups, probably as a result of the surgical debridement required to prepare the wound bed. However, there was no difference in wound area between the groups.

There was a higher prevalence of male patients in our study, and this could be considered a limitation of our study because there may be differences in wound healing between the genders 13,14. This higher male prevalence was expected because the patients in this study were recruited in a trauma center, where the majority of victims are males who were involved in traffic accidents. Still, we observed no baseline difference between the groups with regard for gender, and the male prevalence was the same in both groups.

Another limitation that could be noted is the lack of blinding, which raises the possibility of investigator bias. Patient allocation could not be blinded because the two devices are clearly different from the point of view of both the researchers and the patients. We minimized this risk by blinding the two evaluators who used ImageJ software to measure the wound area and determine the percentage of area with granulation tissue.

The total number of wound treatments analyzed in the study did not reach the estimated sample size, mainly because we had difficulties with the supply of required materials in our public hospital. Additionally, some patients were first admitted in our hospital (a referral center for trauma) but were later transferred to another hospital, resulting in loss to follow-up. However, even if the length of the study had been extended to increase the sample size, it would not have influenced the primary outcome result. The similarity between the two evaluated methods of performing NPWT has also been reported by multiple authors in different countries 2,6,10,11.

The USP method of performing NPWT is non-inferior to the VAC System for treating complex traumatic injuries.

## AUTHOR CONTRIBUTIONS

Kamamoto F was responsible for the study design, data collection and interpretation, manuscript drafting, and approval of the final version of the manuscript for publication. Lima AL was responsible for the study design, data collection and interpretation, and approval of the final version of the manuscript for publication. Mattar-Junior R was responsible for the study design, data collection, approval of the final version of the manuscript for publication. de Rezende MR, Leonhardt MC, Kojima KE and dos Santos CC were responsible for the data collection and analysis, and approval of the final version of the manuscript for publication.

## Figures and Tables

**Table 1:** Demographics and Clinical Characteristics.

Variable	USP (n=19)	VAC (n=32)	*p*
Mean age (years)	34.7±15.1	33.4±16.1	0.603
Gender, male (%, n)	84% (16)	90% (29)	0.659
Trauma by traffic accidents*	94%	84%	0.392
Anatomical region (inferior limb)	93.8%	94.7%	0.885
Initial area (cm^2^)	80±55	105.7±106.3	0.711

* Car or motorcycle collision or pedestrian run over.

**Table 2:** Cost of dressings (US$/day).

Variable	USP (n=18)	VAC (n=32)
Mean cost per dressing	US$ 8.22	US$ 513.29
Mean treatment cost	US$ 15.15	US$ 872.59

**Table 3:** Comparative cost of dressings (US$/day).

	VAC initial	VAC post	USP initial	USP post
Area	105.71074	93.013	80.00431	73.41
Granulation	38.68743	82.86846	34.03333	87.03500
Cost		2757.4000		47.8947
